# Anticipating Economic Market Crises Using Measures of Collective Panic

**DOI:** 10.1371/journal.pone.0131871

**Published:** 2015-07-17

**Authors:** Dion Harmon, Marco Lagi, Marcus A. M. de Aguiar, David D. Chinellato, Dan Braha, Irving R. Epstein, Yaneer Bar-Yam

**Affiliations:** 1 New England Complex Systems Institute, Cambridge, MA, United States of America; 2 Universidade Estadual de Campinas, Campinas, SP, Brazil; 3 University of Massachusetts Dartmouth, Dartmouth, MA, United States of America; 4 Brandeis University, Waltham, MA, United States of America; IFIMAR, UNMdP-CONICET, ARGENTINA

## Abstract

Predicting panic is of critical importance in many areas of human and animal behavior, notably in the context of economics. The recent financial crisis is a case in point. Panic may be due to a specific external threat or self-generated nervousness. Here we show that the recent economic crisis and earlier large single-day panics were preceded by extended periods of high levels of market mimicry—direct evidence of uncertainty and nervousness, and of the comparatively weak influence of external news. High levels of mimicry can be a quite general indicator of the potential for self-organized crises.

## Introduction

The 2007–2008 financial crash led to renewed interest in the development of models capable of predicting and mitigating the severity of future crises, but the question of whether it is possible to predict financial crises has a long history. Methods proposed include the multiple-indicator multiple-cause model [1], multiple-correlations and graphical techniques [2], multiple anomalous indicator thresholds [3], a probit-based model [4], the credit gap [5], the asset price moving average [6], the leverage of the banking sector [7], the absorption ratio [8], and the Hindenburg Omen [9]. Still, there is a better track record of predicting the spatial spread of a crisis than its timing [10]. Recent strategies characterizing market events variously described as multidisciplinary, complex systems, or econophysics approaches include network topologies [11–18], multi-agent models [19–23], response networks [24–28], invariance across many scales [29–34], and the relationships of market behavior with internet search [35]. The literature generally uses volatility and the correlation between stock prices to characterize risk [11, 15–17, 36–40]. These measures are sensitive to the magnitude of price movement and therefore increase dramatically when there is a market crash. Studies find that, on average, volatility increases subsequent to price declines, but do not show that higher volatility is followed by price declines [41–44].

In recent years there has been a scientific focus on network topology models of a wide variety of complex systems. Frequently, sparse networks with heterogenous node connectivities are observed, and the dynamics of those connectivities are of importance [45, 46]. To identify signatures of risk, financial networks [12, 47–49] have been defined primarily from correlational properties of prices [11–18]. For example, Bonnanno et al [14] show that a spanning tree description of correlations [12] shrinks topologically and has distinct power-law exponents during “crash” periods. Harmon et al [16] analyze the correlation network to reveal the changing relationships among the financial, real estate, technology and basic materials sectors from 2000 to 2008, and show that the financial sector propagates the crises between the others, suggesting that firewalls between services for different sectors would reduce systemic risk without hampering economic growth. This finding was reinforced [18] by measures of the role of one stock price on the correlations between others, and subsequent studies [17] further investigated the dynamics of topological properties during the crises period.

Agent based models of the trading strategies of market participants are also used to characterize market behavior. Simulations often consider two groups of market participants: ‘fundamentalists’ and ‘noise traders’ [21–23]. Fundamentalists consider the value of the asset, while noise traders also consider the dynamics of prices, which may result in herding. Simulations suggest [22] empirical volatility clustering and power-law scaling [11, 50, 51] emerge as traders move from one group to another.

A third approach, used in this paper, focuses on modeling the collective dynamics of market prices [24–26]. This approach to multiscale characterization of complex systems derives from prototypical analyses of dynamical processes [52] using concepts from phase transition theory [53]. Such models represent external forces and interactions that result in collective behaviors. Another example of such a model is a study of the dynamics of failure and long term recovery of economic systems [27]. Market collective modes have also been explored by Bury [28] using an information theory based study of increases in coupling of reversals of market indices, though finding that predictive utility of this coupling is limited. Our approach is distinct in focusing on the market collective modes in relation to intermittent market crashes.

Our model focuses on two characterizations of market price behavior. The first is that of traditional economic theory, which considers market prices to reflect perceptions of fundamental value, and therefore changes in market prices to be driven by news, i.e., new information that changes perceptions of fundamental value. The second, from complex systems science, of internal self-reinforcing behaviors that can also give rise to price dynamics. Incorporating both, we construct a universal representation of the largest scale system behavior when there is both external and mutual influence. The resulting network response model was previously introduced by [24, 25] who provided the exact statistical distributions of the fraction of elements that move in the same direction at the same time (the “co-movement” fraction) for fully connected networks of arbitrary size. The results are also excellent approximations for other network topologies, including random, regular lattice, scale-free and small-world networks, when normalized to take into account the effect of topology on coupling to the environment. This model and its analytical results can describe a wide variety of networked systems, from Glauber dynamics of the Ising model [24, 25] and evolutionary dynamics of replicate numbers in population genetics [54, 55] to opinion dynamics on social networks reflecting conformity and non-conformity in social systems [56]. Here, we apply the network response model for financial markets.

Across the parameter space of the model, the system behavior demonstrates an order-to-disorder phase transition, with the appearance of a transition point to collective order in the coupling between the elements as the strength of interactions between them increases. There are two parameters of the model; together they control the relative importance of internal and external causes, and the relative proportion of positive and negative external influences. We show that as we move around in the parameter space, three different types of behavior are observed: an “up” phase corresponding to skewed distributions with a high fraction of stocks that move up (positive price movement); a “down” phase corresponding to skewed distributions with a low fraction of stocks that move up (negative price movement); and a region corresponding to bimodal distributions in which two symmetry breaking phases may exist due to slow dynamical switching between them, i.e. hysteresis. The spontaneous emergence of phase switching (flipping) phenomena corresponds to a first order phase transition. The *critical value* of this model, the transition between disordered and ordered states, is a unique state with a flat distribution.

The model is relevant to dynamics of multiple equities, rather than individual stock behaviors. The behavior can still be considered to arise from trading agents, and might be represented by networks of influence between them. However, many of the details are not relevant and are thus abstracted into aggregate behavior in our analysis. Thus, for example, as indicated above, the structure of the network does not change the behavior, and unlike trader agent models the behavioral rules of our price agents need not differ. The natural behavior that we find is a transition between independent and collective action, the latter of which can be identified with panic. We are thus able to identify a measure of collective panic and use it to predict financial crises that follow when panic occurs.

In sociology [57–60], panic has been defined as a collective flight from a real or imagined threat. In economics, bank runs occur at least in part because of the risk to the individual from the bank run itself—and may be triggered by predisposing conditions, external (perhaps catastrophic) events, or even randomly [61, 62]. While market behavior is often considered to reflect external economic news, empirical evidence suggests that external events are not the only cause of market panics [63]. Although empirical studies of panic are difficult [64–66], efforts to distinguish endogenous (self-generated) and exogenous market panics from oscillations of market indices have met with some success [30–34], though the conclusions have been debated [67–70].

Linking concepts of panic to our influence model, we identify mimicry of panic as mutual influence. We test this empirically against the daily extent of co-movement. The extent of such co-movement may be large even when price movements are small, so we consider co-movement to be the collective behavior that is characteristic of panic and nervousness. Thus, rather than measuring volatility or correlations, we measure the fraction of stocks that move in the same direction. Remarkably, the distributions predicted for the behavior of the model are robustly confirmed by successful testing on real-world financial data, covering the recent economic crisis as well as earlier market dynamics. We use the co-movement data to evaluate whether the recent market crisis and historical one-day crashes are internally generated or externally triggered. Over the period of our analysis the real world behavior narrowly adopts only the balanced positive and negative news one-dimensional subspace of the parameter space. We find that the critical point with high levels of co-movement, i.e. panic, is found to uniquely identify the 2008 market crash. Since the critical point is unique, no model parameters are adjusted to obtain this correspondence, so this can be considered as a zero parameter theory of the financial crisis. Moreover, a measure of co-movement increases well before one day market crashes, and there is significant advance warning to provide a clear indicator of an impending crash. Increasingly panicky behavior is thus an early warning sign of each market crash as a ‘critical transition’ [71, 72]. Our predictive performance is exceptional—it anticipates the largest one-day crashes over 25 years, with no false positives or negatives. We compare our results with other possible predictors of market crises: volatility, correlations and covariance between equity prices. We modify the traditional direct use of these indicators by implementing thresholds of sharp increases, and find that this approach results in statistically significant predictive utility. Of these indicators, volatility and correlations, the most common used risk predictors, provide the least predictive ability with three errors and four correct predictions, covariance is a comparatively better predictor with only one error, and our model provides the best predictive utility with no errors. An earlier account of the main results of our analysis is available [26].

## Results

We describe our results beginning from empirical observations, motivate the construction of the quantitative model in the context of prior economic theory, and compare the results of analytic solution of the model with the empirical observations.

**Fig 1:**
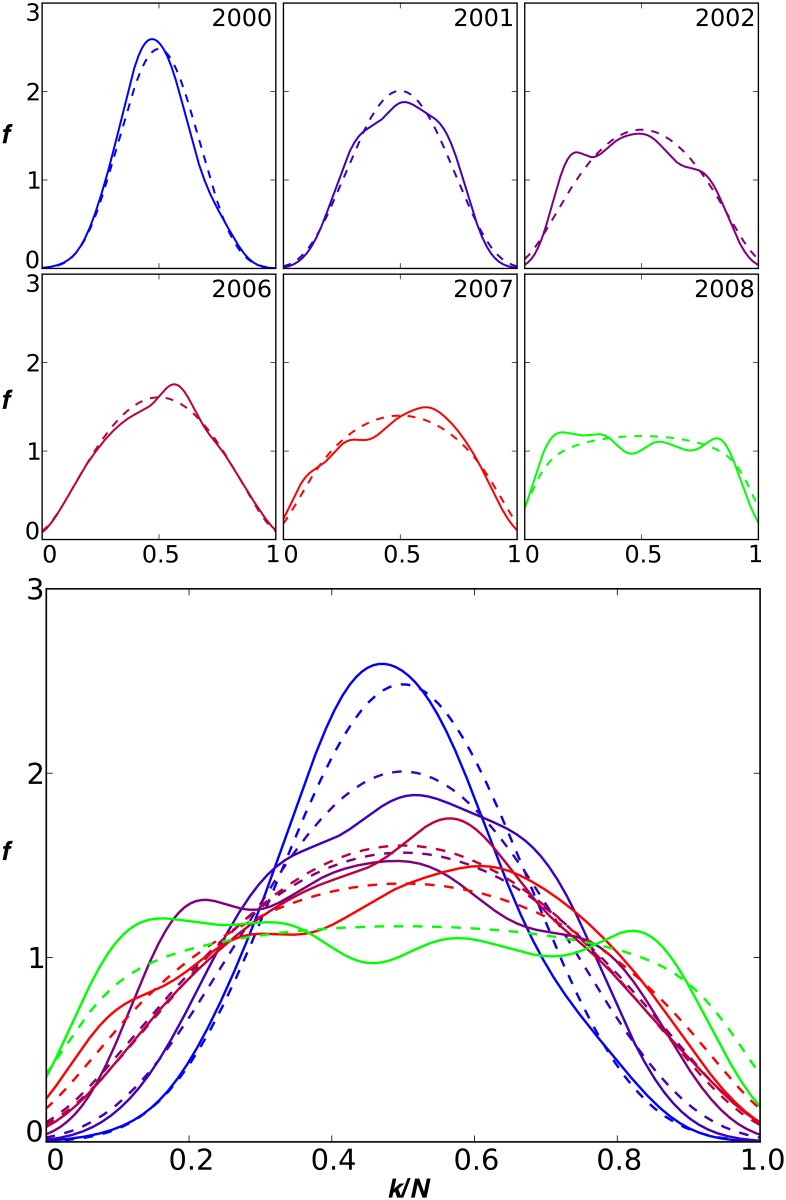
The co-movement of stocks. Plotted is the fraction of trading days during the year (*f*, vertical axis) in which a certain fraction of stocks (*k*/*N*, horizontal axis) moved up. Empirical data are shown (solid lines) along with one-parameter theoretical fits (dashed lines) for the years indicated. Three years are omitted that do not differ much from the year immediately preceding and after them. Bottom panel combines all of the years shown. Stocks included are from the Russell 3000 that trade on the NYSE or Nasdaq. Curves are kernel density estimates with Gaussian kernels (*σ* = 0.06). Fits pass the *χ* ^2^ goodness-of-fit test (the deviation of the data from the theoretical distribution is not statistically significant at the 25% level).

We consider the “co-movement” of stocks over time by plotting the number of days in a year that a particular fraction of the market moves up (or down). Intuitively, if substantially more or less than 50% of the market moves in the same direction, this represents co-movement. As shown in Fig 1, the results indicate that in 2000, the curve is peaked near 1/2, so that approximately 50% of stocks are moving up or down on any given day. Over the decade of the 2000s, however, the curve became progressively flatter—in 2008 the likelihood of any fraction is almost the same for any value. The probability that a large fraction of the market moves in the same direction, either up or down, on any given day, increased dramatically. Such high levels of co-movement may manifest the collective behavior we are searching for.

To quantitatively describe co-movement, we start from a behavioral economics model of a single stock that describes trend-following “bandwagons.” It has been shown that investors can benefit from trend-following [73–76]. Moreover, there is no need for the change to be based upon fundamental value for it to provide benefit to the investors [73, 74]. When individuals observe that a stock increases (decreases) in value, and choose to buy (sell) in anticipation of future increases (decreases), this self-consistently generates the desired direction of change. Such a “bandwagon” effect can undermine the assumptions of market equilibrium. We hypothesize that this trend-following mimicry across multiple stocks can cause a marketwide panic, and we build a model to capture its signature. We assume that investors in a stock observe three things, the direction of their stock, external indicators of the economy, and the direction of other stocks. The last of these is the potential origin of self-induced market-wide panic.

To model the co-movement fraction, we represent only whether a stock value rises or falls. This enables us to directly characterize the degree to which stocks move together and not how far they move at any particular time. Stocks are represented by nodes of a network and influences between stocks by links between nodes, an appropriate representation for market analysis [11, 15, 38, 48]. To represent external influences, we add nodes that influence others, but are not themselves influenced, i.e. “fixed” nodes. The number of fixed nodes influencing in a positive direction is *U* and the number influencing in a negative direction is *D*. The effective strength of the positive and negative external influences is given by the number of these nodes. Thus, we consider a network with *N* + *D* + *U* nodes. Each node has an internal state which can take only the values −1 or +1, representing whether the stock value increases or decreases on that day, or, for fixed nodes, whether news is positive or negative. We assume that the *N* nodes (stocks) change their internal state according to a dynamical rule: At each time step a random free node is selected and its state is updated with probability 1 − *p* by copying the state of one of its connected neighbors, chosen at random from all nodes; and with probability *p* the state remains the same. The *D* nodes remain fixed in state −1 and *U* nodes in state +1. Copying the state of a connected dynamic node represents mutual influence, while copying from a fixed node represents the influence of external news. Analytically extending *D* and *U* to non-integer values enables modeling arbitrary relative strength of external to internal influence (see Methods and [24, 25]). We note that in this model external influences of opposite types do not cancel; instead larger *U* and *D* reflect increasing probability that external influences determine the returns of a stock independent of the changes in other stocks. This is the conventional view that news is responsible for the market behavior. The model assumes that there are many news items and that over the period in question the news is persistent in its proportion of positive and negative values though it varies in the way it influences individual stock values. Periods of consistently good news would be represented by *U* greater than *D*, bad news by *D* greater than *U*.

As described in the introduction, we have previously proposed this model as a widely applicable theory of collective behavior of complex systems, prior to comparison with economic data. [24, 25] Successful matching to data will be a confirmation of the universality of this theory.

The behavioral model can be solved exactly for a fully connected network (see Methods). We obtain the probability of a co-movement fraction:

$$f(k/N)=\frac{\left(\begin{array}{c} U+k-1\\ k \end{array}\right)\left(\begin{array}{c} N+D-k-1\\ N-k \end{array}\right)}{\left(\begin{array}{c} N+D+U-1\\ N \end{array}\right)}$$ (1)

where N is the number of stocks, *k* is the number of stocks with positive returns and $\left(\begin{array}{c} n\\ k \end{array}\right)$ are binomial coefficients. The behavior is controlled by the strength of external stimuli, U and D, compared to the strength of interactions within the network, and the relative bias of the external influence toward positive, U, or negative, D, effects. When interactions are weak compared to external forces (*D*, *U* > > 1), the distribution is essentially normal. When internal interactions are strong (small *D*, *U*), the distribution is neither normal nor long-tailed. Instead it becomes flatter, becoming exactly flat at the critical value (*D* = *U* = 1), where the external influences only have the strength of a single node. Analytic continuation allows *U* and *D* to be extended to non-integer values. There are three parameters of the distribution, *D*, *U*, *N*, but the third is fixed to the number of stocks. We can compare this to the binomial or normal distributions, which are specified by two parameters, the average and standard deviation. The distribution we obtain has a wider range of behaviors, and the normal distribution arises as a limiting case.

If we consider a more complete model of influences, in which investors of one stock only consider specific other stocks as guides, we have a partly connected network. We have studied the dynamics of such networks analytically and through simulations, and the primary modification from fully-connected networks is to amplify the effect of the external influences (see Methods and [24, 25]). As the links within the network are fewer, the network can be approximated by a more weakly coupled, fully connected network, with a weakening factor given by the average number of links compared to the number of possible links. Similarly, if only a subset of the external influences are considered relevant for the return of a specific stock, the relative strength of the external influences can be replaced by weaker, uniform external influences. Otherwise, for many cases, the shape of the distribution is not significantly affected. The model’s robustness indicates a universality across a wide range of network topologies, suggesting applicability to real world systems.

Compared with recent empirical market data in Fig 1, the model fits remarkably well. A Gaussian model fits the early years, less well in the final years, and does not fit the data of 2008. The good agreement of our model is obtained with equal up and down influences, *D* = *U*, which is the only adjustable parameter. This implies that whether the market value is trending up or down, or has large one day drops, over a period of a year co-movements occur symmetrically in both up and down directions. Model parameter values for the distributions in Fig 1 are given in Table 1.

**Table 1:** Model parameter values used to generate the distributions in Fig 1. Empirically, we find that stock return distributions are symmetric, reducing our model to only one free parameter, *D* = *U*. Similar results are obtained using direct fits and by using the standard deviation of the distribution (see Methods).

Year	*U* = *D*
2000	5.79
2001	3.66
2002	2.21
2006	2.32
2007	1.77
2008	1.24

The economic crisis period’s flat distribution corresponds to *D* = *U* = 1. This is the critical value of the model where external influences are very weak compared to the influences among stocks as a whole. By contrast, predominantly negative effects, *D* > *U*, would manifest as a distribution whose mean is shifted to the left. Thus, rather than negative news, uncertainty and collective mimicry led to a self-organized crash.

**Fig 2:**
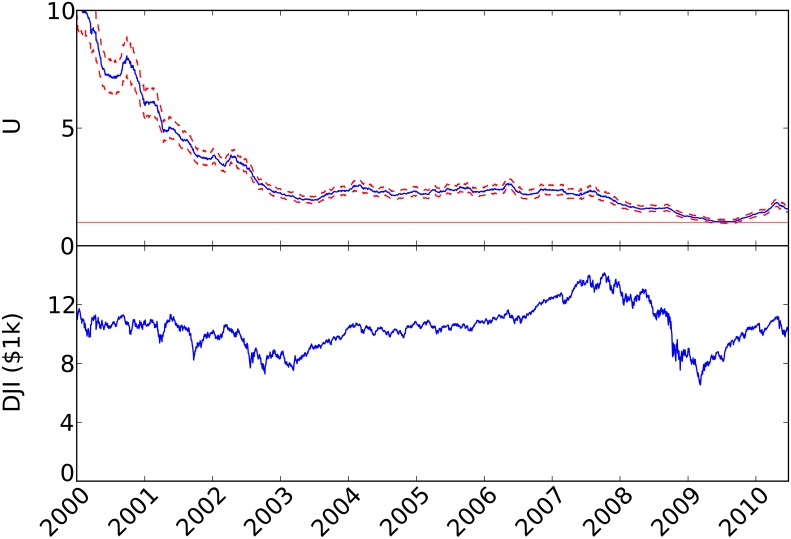
Model parameter (top panel) and the Dow Jones Industrial Average (bottom panel) for the period 2000–2010. Estimates of the model parameter are shown at the end of the year-long period for which *U* was estimated. Sampling error estimates are drawn at ±1 standard deviations. Positive-return distributions are computed from the daily returns of stocks of the Russell 3000.

The flattening of the stock market distribution may serve as a measure of market vulnerability to panic, and the projection of a flat distribution observed in the economic crisis can be used as an early warning signal. Fig 2 shows the empirical results of the single parameter *U* (= *D*) from 2000–2010. We note that the average used for the value at any point of time is from the period of 12 months prior to that time in order to evaluate the predictive ability. A significant drop occurred in the 2000–2002 period, followed by a plateau that declined gradually beginning in mid-2007 until it hit the critical value at *U* = 1. This suggests the market was vulnerable well before the financial crisis, and the gradual decrease before the crisis suggests that the crisis could have been anticipated.

**Fig 3:**
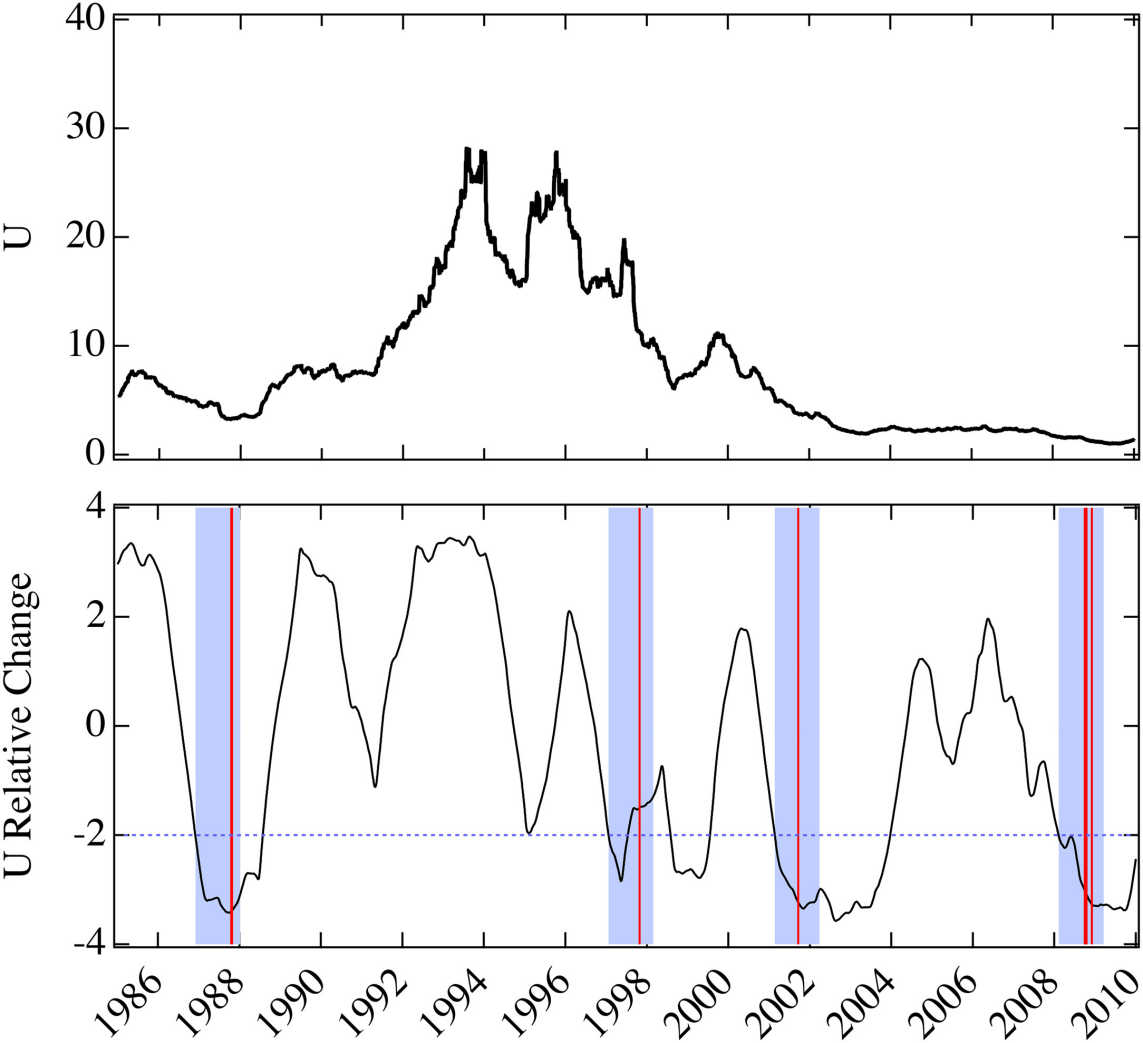
Annual relative change in model parameter *U* for the period 1985–2010. Top panel is the same as in Fig 2 for the period 1985–2010. Bottom panel is the annual change of *U* as a fraction of its standard deviation computed over the previous year. Of the twenty largest percentage drops of the Dow Jones Industrial Average, eight are in the displayed time period: 10/19/1987, 10/26/1987, 10/27/1997, 9/17/2001, 9/29/2008, 10/9/2008, 10/15/2008, 12/1/2008 (vertical red lines). Four year-long windows (shading) follow two standard deviation drops in the model parameter after periods of increase. Such an analysis generates 4 true positives and no false positive.

In order to evaluate more broadly the predictive ability of the model, we consider the period from 1985–2010 (Fig 3). While there was no other financial crisis of comparable magnitude to the current one, drops in the model parameter *U* anticipate large drops of the Dow Jones Industrial Average (DJI). The bottom panel of Fig 3 shows the (annual) change in the model parameter as a fraction of the standard deviation computed over the preceding year. Of the all-time twenty largest single-day percentage drops of the DJI, eight are in the displayed time period [77], proximate to Black Monday [78], the Asian market crisis [79], September 11, 2001, and the recent financial crisis of 2011. A simple signature pattern precedes the drops by less than a year: after a period of positive change, a large drop occurs in the parameter *U*, greater than twice the standard deviation computed over the preceding year. This pattern identifies four year-long windows in which occur the eight largest percentage drops of the DJI within the last 26 years. The performance of the predictive pattern is exceptional (*p* < 0.00007 for four non-overlapping, year-long windows, see Methods).

Two questions might be asked to evaluate the signature robustness. First, the pattern is nearly matched in 1995 when the change of the parameter as a fraction of the standard deviation drops to below −1.67 in April, 1995, but this near match is not followed by a large drop in the DJI within the year. Secondly, the drop in the DJI on September 17, 2001, on the trading day immediately following September 11, 2001, appears to have a direct external cause, and therefore we might not consider the intrinsic stability of the market as predictive, though we do not exclude the possibility that a drop would have occurred without the attack. If we interpret the results conservatively, we would eliminate the year 2001 from consideration (*p* < 0.0005), include the near-prediction in 1995 (*p* < 0.0004), or both (*p* < 0.002). Even in this case, there is strong predictive success.

Our prediction of the event on September 17, 2001 was also obtained by Hurst time series analysis [80], and our work provides additional evidence that this event was not solely a reaction to the events of September 11, but largely reflected intrinsic market dynamics. On the other hand we do not predict an event for 2003. This is to be contrasted with the predictions by others that did not come true [81]. However, we do find a significant drop in *U* prior to that time, suggesting increased vulnerability. It appears that two events conspire to prevent the crash. First, the increase in mimicry leveled off before the systemic instability threshold. Moreover, following the smaller crash on September 17, 2001 there was no actual recovery of the market dynamics, which continued to be vulnerable, but without a crash, until 2007. Our result that increased mimicry *anticipates* panics is also distinct from debates about the origins of higher correlations that *follow* crises [82–84].

## Robustness of the analysis

**Fig 4:**
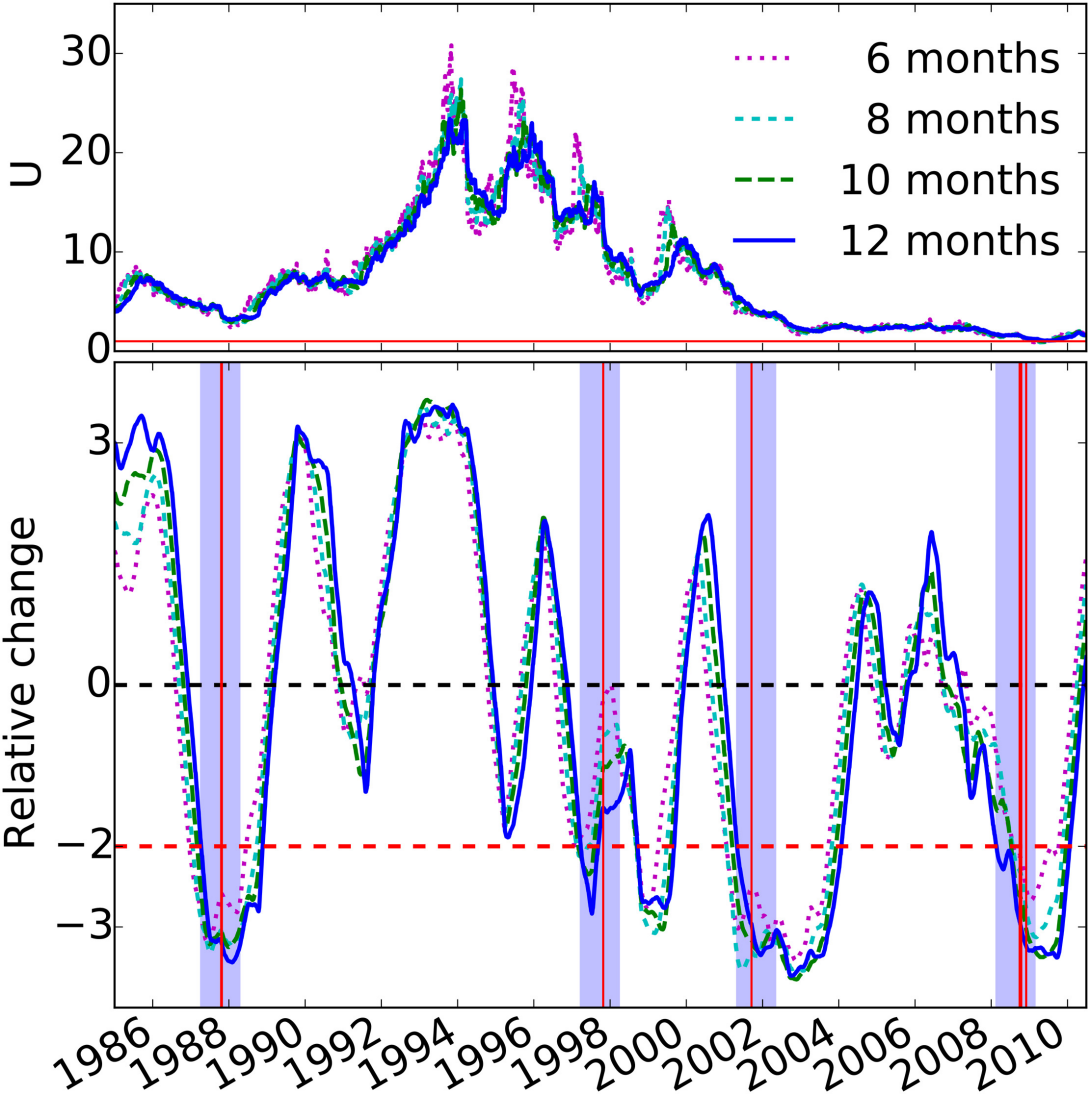
Model parameter *U* (top panel) and relative change in model parameter (bottom panel) for the period 1985–2010, and for varying values of the sliding time window. Similar to that of Fig 3, but for sliding time windows of 6, 8, 10, and 12 months. The relative change of *U* is based on the window-long period for which *U* was estimated. For all cases, four year-long intervals that follow two standard deviation drops in the model parameter overlap with the largest percentage drop events displayed by the vertical red lines.

**Fig 5:**
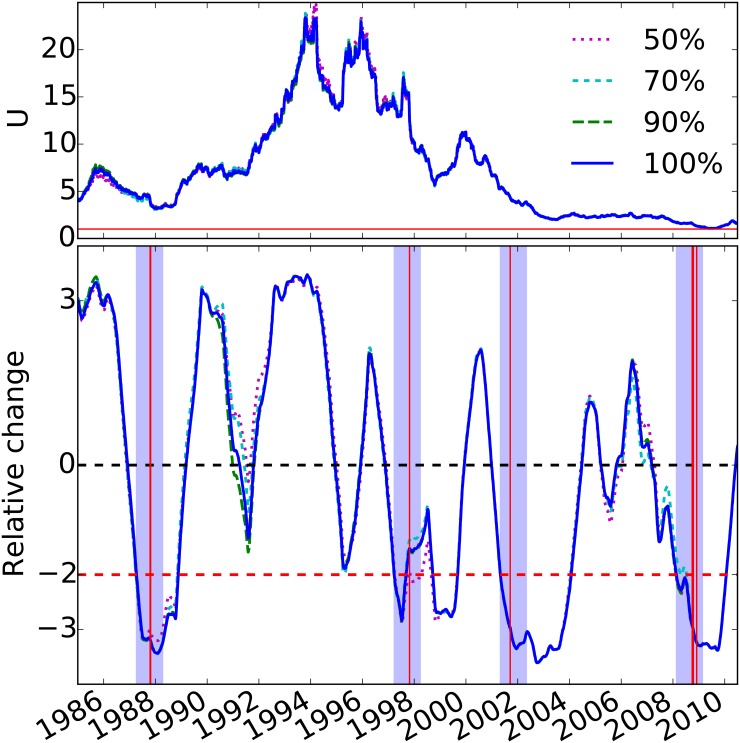
Model parameter (top panel) and relative change (bottom panel) in model parameter *U* for the period 1985–2010, for varying subsamples of stocks. Similar to that of the top panel of Fig 3, but for different subsamples of stocks of 50%, 70%, 90%, and 100% of the entire sample used in Fig 3. Positive-return distributions are computed from the daily returns of stocks included in the corresponding subsamples. The original analysis is robust, with four true positives, i.e., no false positives or negatives, for the 70%, 90%, and 100% subsamples. One false positive is introduced for the 50% subsample due to the reduced sample sizes.

We test the robustness of our results in two different ways. First, we vary the size of the sliding window used to estimate the parameter *U* and the corresponding relative change, as shown in Fig 4. Second, we examine the effect of the size of the sample of stocks, used to compute the co-movement fraction, on the estimated value and relative change in model parameter *U*, as shown in Fig 5. In both cases, we find that our results and the accuracy of the model’s predictability are robust.

## Comparison with standard measures

We compare the predictions of our model to conventional indicators of systemic risk that include volatility, covariance and correlations:

$$\begin{array}{c} \text{volatility:}\;\;\sigma_{x}=\sqrt{\text{Var}(x)}=\sqrt{E\left[x^{2}\right]-E{\left[x\right]}^{2}}\\ \text{covariance:}\;\;\text{cov}(x,y)=E[xy]-E[x]E[y]\\ \text{correlation:}\;\;\rho \left(x,y\right)=\frac{\text{cov}(x,y)}{\sqrt{\text{var}(x)\text{var}(y)}} \end{array}$$ (2)

where *E*[…] is the expectation value and (*x*, *y*) the time series of two assets.

We focus on their annual change relative to their standard deviation, from 1985 to 2010. We find that they all have some predictive ability with respect to the biggest percentage drops of the Dow Jones Industrial Average. However, our model parameter is the only one that can predict all the events, with no false positives or negatives.

**Fig 6:**
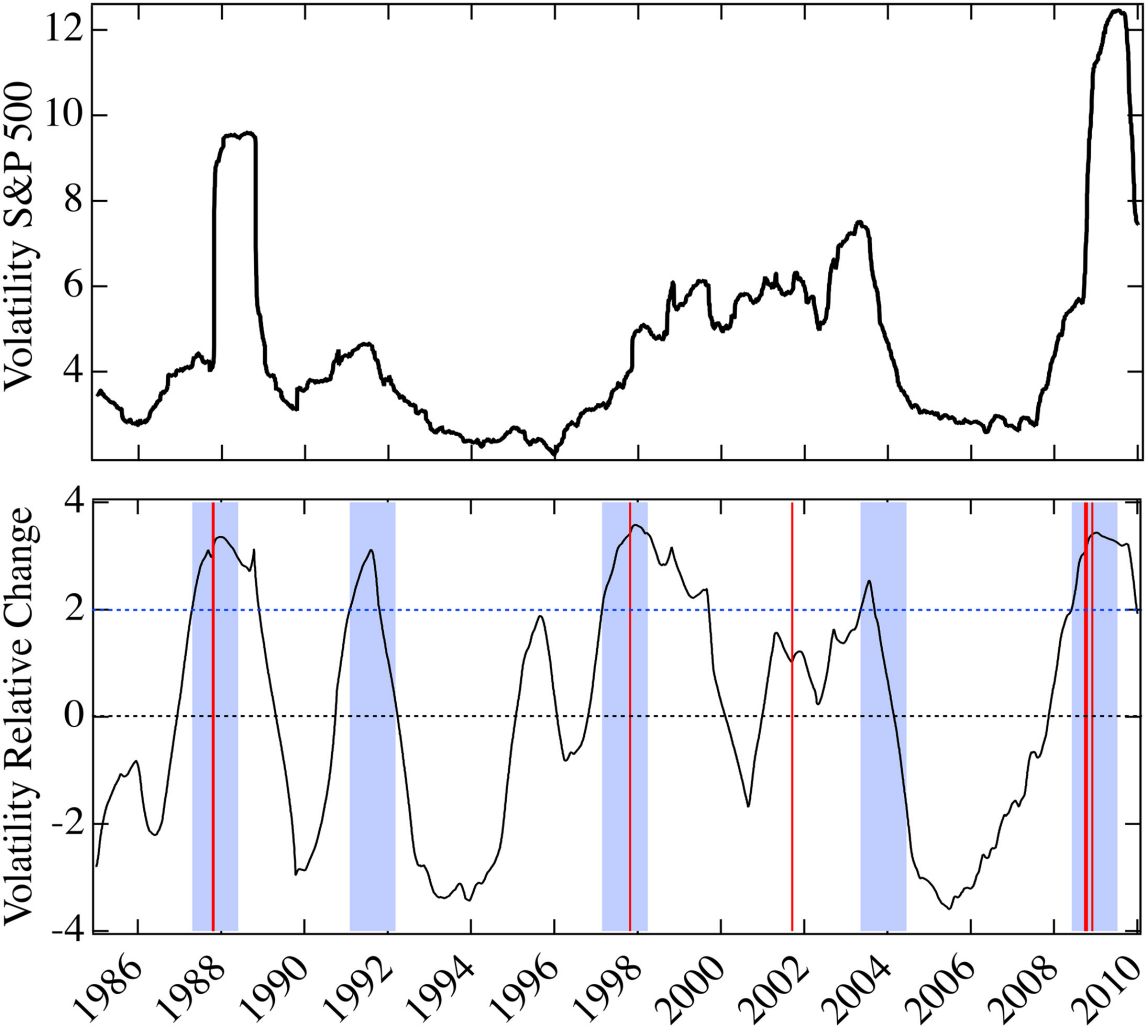
Annual relative change in volatility for the period 1985–2010. Top panel is the volatility of the S&P 500 index, from 1985 to 2010, averaged over the previous year. Bottom panel is the annual change of volatility as a fraction of its standard deviation computed over the previous year. Four year-long windows (shading) follow two standard deviation increases in volatility greater than twice the standard deviation from one year earlier, after periods of decline. One day crashes are as in Fig 3 (vertical red lines). This indicator generates 3 true positives, 2 false positives and 1 false negative.

**Fig 7:**
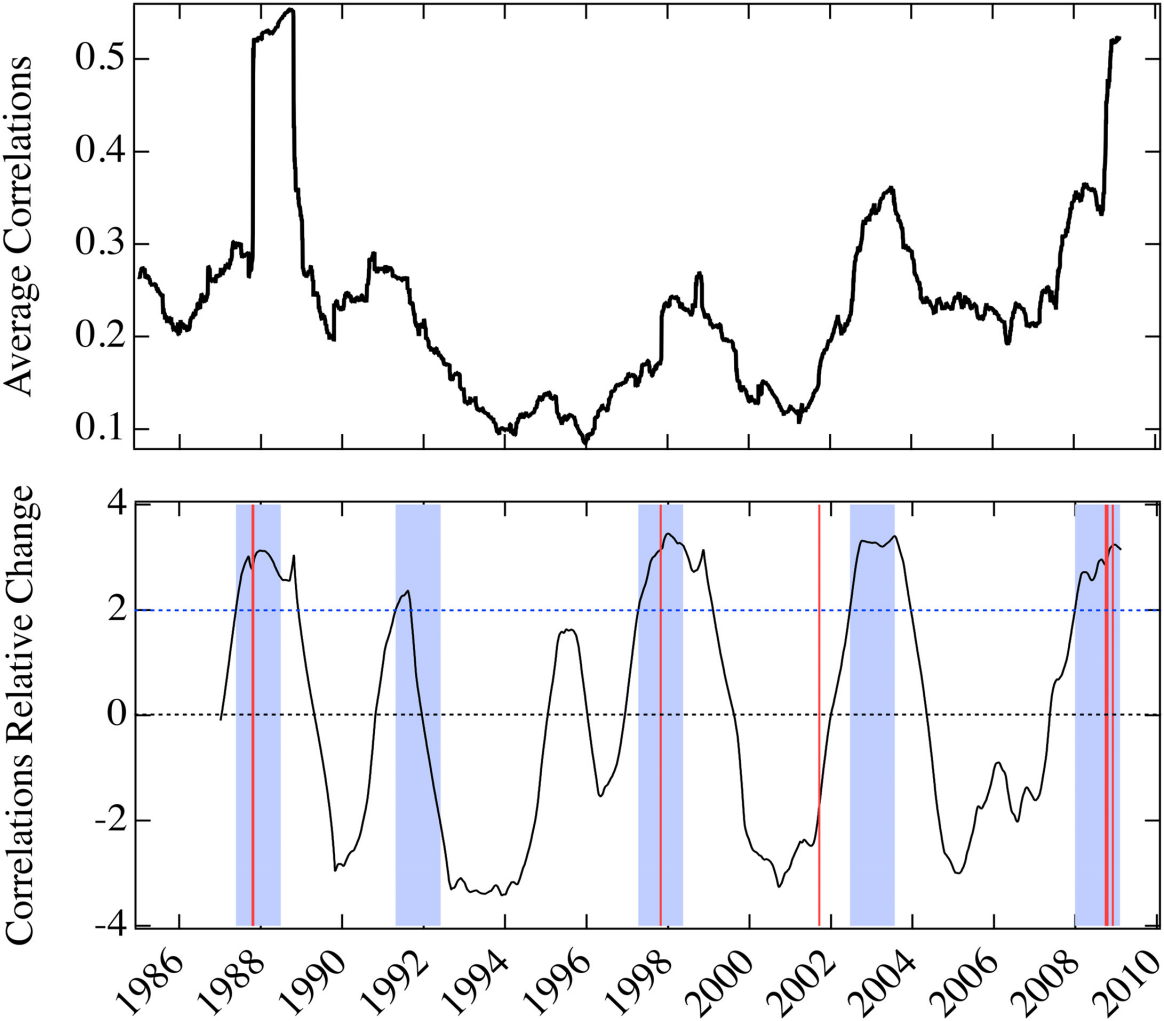
Annual relative change in correlations for the period 1985–2010. Top panel is the average Pearson’s correlation coefficient between price returns of the underlying components of the S&P 500 index, from 1985 to 2010. Bottom panel is the annual change of the average correlations as a fraction of its standard deviation computed over the previous year. Four year-long windows (shading) follow two standard deviation increases in the average Pearson’s correlation greater than twice the standard deviation from one year earlier, after periods of decline. One day crashes are as in Fig 3 (vertical red lines). This indicator generates 3 true positives, 2 false positives and 1 false negative.

**Fig 8:**
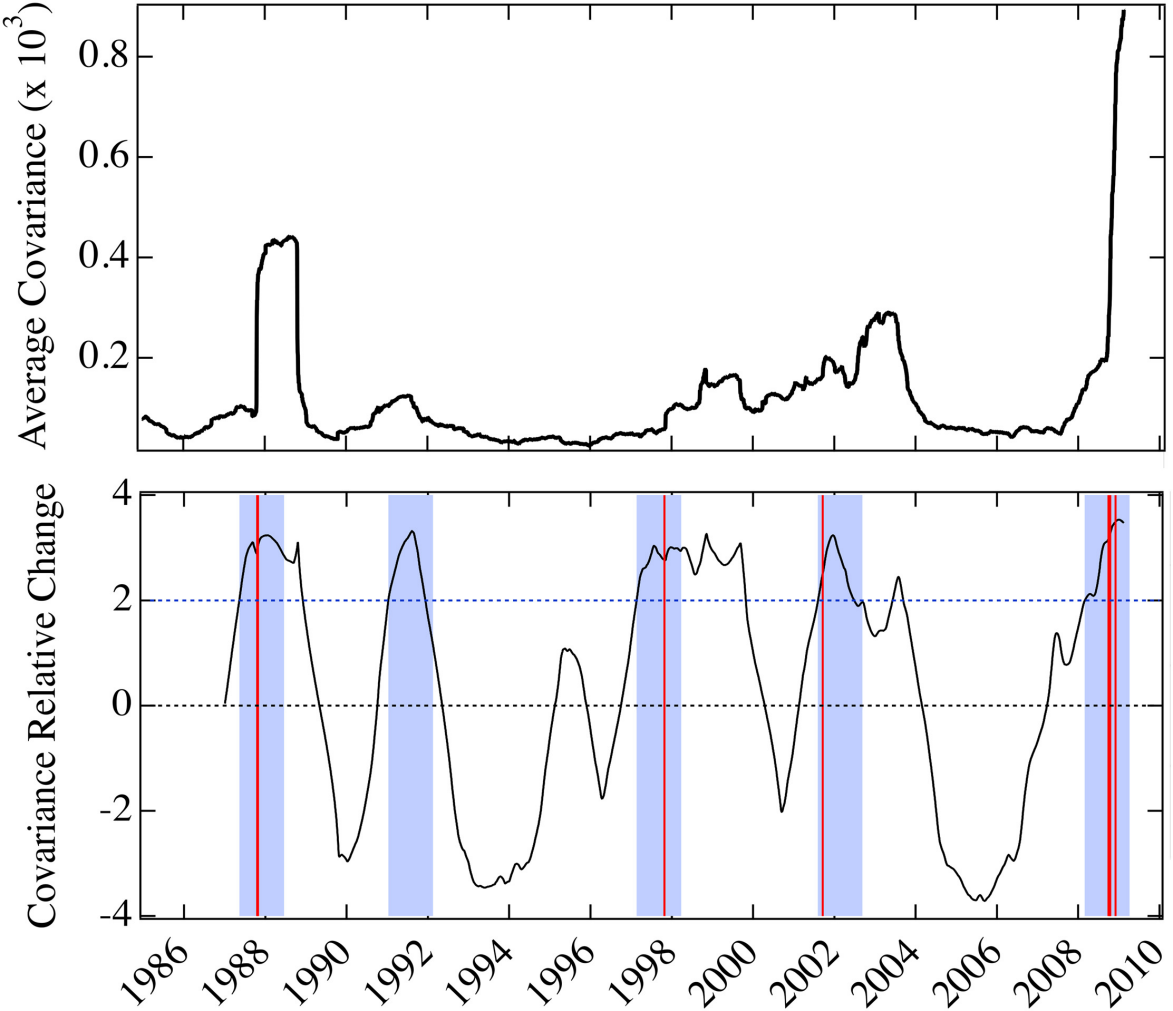
Annual relative change in covariance for the period 1985–2010. Top panel is the average covariance between price returns of the underlying components of the S&P 500 index, from 1985 to 2010. Bottom panel is the annual change of the average covariance as a fraction of its standard deviation computed over the previous year. Four year-long windows (shading) follow two standard deviation increases in the average covariance greater than twice the standard deviation from one year earlier, after periods of decline. One day crashes are as in Fig 3 (vertical red lines). This indicator yields 4 true positives and 1 false positive.

In Figs 6–8, we show the predictive ability of the three measures between 1985 and 2010, which should be compared with Fig 3 (the analogous treatment of our model parameter *U*). In all the figures, the top panels display the measure, and the bottom panel shows the annual change as a fraction of their standard deviation computed over the preceding year, the *relative change*:

$${\text{R}}_{t}=\frac{X_{t}-X_{t-365}}{\sigma_{t,t-365}}$$ (3)

where *X* denotes one of the measures: volatility of the S&P 500 index, average covariance, average Pearson’s correlations of the S&P 500 underlying components, and our model parameter *U*. Our objective is to find the change of the measure *X* anticipating the largest market drops. As a signature, i.e., a positive prediction, we choose a large increase in the measure *X* (decrease for our parameter *U*) greater than twice the standard deviation from one year earlier. This identifies a year-long window within which the crash is supposed to occur (blue shading in Figs 3 and 6–8). Of the twenty largest percentage drops of the Dow Jones Industrial Average, eight fall in this time period, in the vicinity of Black Monday, the Asian market crisis, 9/11, and the 2007–8 financial crisis. When one of the drops falls in a blue region the prediction for the that year is a true positive (TP), if it falls outside a blue region the prediction for that year is a false negative (FN), and if a blue region does not contain an event the prediction for that year is a false positive (FP). The increase (or drop, for our parameter *U*) is not considered a prediction if the value of the relative change of *X* has not changed sign with respect to the previous increase (or drop). We find that the volatility, covariance, and correlation indicators all have statistically significant predictive capability, with the volatility and correlations (the most common used risk predictors) providing the least predictive ability, covariance is a comparatively better predictor, and our model parameter *U* provides the best predictive utility. Results for the four indicators are summarized in Table 2.

**Table 2:** Comparison of predictors of market crises. For each predictor (classifier), the table reports the number of true positives (TP), false positives (FP), false negatives (FN), and the probability of obtaining this performance assuming a random predictor.

Indicator	TP	FP	FN	p-value
Volatility	3	2	1	0.014
Correlation	3	2	1	0.014
Covariance	4	1	0	0.00033
*U*	4	0	0	0.000067

The parameter *U* from our model outperforms the other predictors in standard metrics, including Precision, Recall, Accuracy, F-score, and Matthews correlation coefficient [85]. To clarify the predictive power of our model relative to the other indicators, we define a statistical “goodness of fit” metric (Table 2). Let there be *n* years of which *r* are ‘crisis’ years and *s* = *n* − *r* are ‘non-crisis’ years. For a binary classifier, let TP be the number of true positives, and FP be the number of false positives. If we assume that the classifier is random, the probability of obtaining the *observed outcome* of the classifier can be shown to be:

$$\text{p-value}=\frac{\left(\begin{array}{c} r\\ \text{TP} \end{array}\right)\left(\begin{array}{c} s\\ \text{FP} \end{array}\right)}{\left(\begin{array}{c} n\\ \text{TP+FP} \end{array}\right)}$$ (4)

The p-value represents a measure of the evidence against the random classifier assumption: the smaller the p-value, the stronger the evidence against the random classifier assumption.

For a perfect classifier, TP = *r* and FP = 0, and thus we obtain from Eq 4:

$$\text{p-value}=\frac{1}{\left(\begin{array}{c} n\\ r \end{array}\right)}$$ (5)

The eight major financial crashes in the time window are clustered in four main events. As shown in Fig 3, our model parameter *U* generates 4 true positives and no false positive, resulting in a p-value of 0.000067, which is the lowest, i.e. most predictive of the measures as shown in Table 2.

If we look more closely at the volatility (Fig 6, top panel), we see that there are smaller increases before some of the crashes, but also that there are such increases even when there are no crashes. This can be made evident using our method of obtaining a signature, i.e. taking the increase over a year of the predictor and dividing it by its standard deviation (see Fig 6, bottom panel). We see that the 1987, 1997, and 2008 crashes are predicted, but there are two false positives in 1991 and 2003 and a false negative in 2001. The false positives reduce the statistical measure of prediction dramatically relative to our method (see Table 2). That the three largest peaks coincide with the crashes is primarily due to the large increase of volatility when the crash occurs and afterwards.

As mentioned previously, the literature does not claim that prediction can be made based upon volatility, even though it is considered a measure of risk. It might be thought that measures of risk should be particularly high before a crash, but this is not what is observed (Fig 6, top panel). The most dramatic property of the volatility is that it increases when a crash occurs, and it stays high thereafter. Here we considered the average over a year, so the impact of the crash on a particular day can be seen for a year after it occurred, but the volatility tends to be higher after a crash even without this effect. There is no strong correlation of high volatility with the period of time before the crash, so its time series cannot be considered to have good predictive ability.

While the corresponding relative change of the average correlations among stocks has a similar performance to the volatility indicator (see Fig 7), the average covariance indicator performs better than volatility and correlations, since it is able to predict all the events (see Fig 8). All these indicators predict a false positive in 1991, which may be related to the Persian Gulf crisis that lasted from August 1990 to January of the following year. Our model parameter, with all crashes anticipated, has no false positives. The 1991 episode demonstrates the predictive ability of our model, derived from its capability to single out exclusively instances in which mimicry is present. The downturn driven by the external negative news of the Persian Gulf crisis, which resulted in a lengthy but not dramatic financial decline, did not result in mimicry and therefore does not lead to a prediction within our model.

In summary, we compared the predictive utility of our signature of panic to other indicators of systemic risk: volatility, correlations and covariance. For each, we calculated the annual change of each indicator relative to their standard deviation, from 1985 to 2010. We found that they all have some predictive utility with respect to the largest percentage drops of the Dow Jones Industrial Average. However, the four indicators were found to behave differently; only for our model parameter *U* the prediction generated all cases correctly, i.e. no false positives or false negatives. Still, each predictor has utility, and it is possible that multiple predictors, used in a composite classifier architecture [86], can provide additional insights for early warning indicators of vulnerabilities and critical tipping points of financial and economic systems. However, given the limited data that is available about large one day crashes additional developments would be needed to motivate such a classifier.

## Discussion

In previous work [24] [25], we provided exact statistical distributions for the dynamic response of influence networks subjected to external perturbations—a problem of great methodological and practical importance. Here, we apply the general analysis of statistical distributions to obtain a measure of collective panic to predict financial crises. In this paper two innovations are presented: 1) the introduction of a single-parameter model that quantifies market mimicry, 2) a new method to identify an upcoming crisis, i.e. considering the annual change of our model parameter relative to its standard deviation. We showed that long periods of high levels of market mimicry preceded the 2007–2008 financial crisis and all the other historical large single-day panics since 1985. During these periods, Keynes’ “animal spirits” of uncertainty and nervousness drove down the stock market prices, which were only weakly influenced by external news. Further support for the predictive capability of our model is provided when comparing its predictions to other indicators of systemic risk, that is, volatility, covariance and correlations between equity prices.

Central to the discussion of panic in the literature [57–60] is the degree to which it reflects external threats that cause each individual to panic, or whether it reflects mimicry with or without external causes. Even when mimicry is important, underlying conditions that imply increased risk can elevate sensitivity and the tendency to mimicry. Underlying conditions in this context may include internal trends such as market bubbles, or external factors such as war, or the financial disruptions that preceded the recent market decline. When panic involves collective action, rather than individual response, precursor fluctuations are likely to exist due to a growing sensitivity to real or random disturbances. Our results suggest that self-induced panic is a critical component of both the current financial crisis and large single day drops over recent years. The signature we found, the existence of a large probability of co-movement of stocks on any given day, is a measure of systemic risk and vulnerability to self-induced panic.

One of the interesting results of our analysis is the empirical relation *D* = *U*, which may reasonably reflect the overall neutrality of news affecting the market on a scale that would result in significant bias of the entire distribution over the period of a year. For news to be biased multiple news items affecting individual stocks across a large fraction of the market would have to occur over the entire year. Even when stock prices trend upwards or downwards over a year, they generally don’t move upwards and downwards consistently from day to day. Thus, over a period of a year, observed on a daily scale, the bias of positive and negative news can be expected to be small. While this is sufficient explanation, it is also possible to strengthen this argument based upon a fundamental economics perspective on market prices. This fundamental perspective considers news to be incorporated into the price of stocks once it happens, and the magnitude of the movement of prices in response to news reflects the financial significance of the news, and is not included in our model. Thus, in this perspective adjustment of prices to news happens immediately and without persistence. For persistently positive shifts to take place additional news that is positive relative to prior positive news is needed. Note that any positive persistence, to the extent that it can be anticipated due to its persistence, is no longer news. Rapid fluctuations in stock prices occur at a time scale that allows for reversals many times in a single day. Consistent price movements in sub-day or multi-day periods due to a single external news event is precluded by profit opportunities due to predictability. Thus, to first order, price changes that occur from day to day may be considered to represent a new sample from the underlying statistical distribution. Updates of stock prices over the period of a day therefore are randomly positive or negative when the external influence is large, and given a large number of news, influences will become exactly 50%/50% upwards and downwards. While it is possible for there to be conditions of persistent positive or negative news, these considerations suggest that the extent of positive or negative news persistence is limited. Indeed, we find that the distribution is largely confined to the parameter sub-space, *D* = *U*, where the various news items are equally likely to lead to positive or negative price movements. When there are mutual influences between stock price movements, fluctuations lead to deviations from 50%/50%, but when *D* = *U*, these fluctuations are also equally likely to be in the positive and negative directions. A decrease in the value of *D* = *U* is a signature of increasing positive and negative fluctuations, which would be inconsistent with negative news dominating the behavior of the system. Such a decrease yields what looks like random reversals of stock prices moving together (large co-movements) rather than reversals of individual stocks. The width of the distribution of daily movements then reflects the extent of co-movement generated spontaneously. This can be interpreted as indicative of uncertainty about the direction of market movement, i.e panicky behavior, when the influence of external news relative to internal influence is sufficiently weak. In the model, larger co-movements occur when there are fewer external nodes whose influence would cause independent movements of nodes.

The reported results and methods have several potential applications. The primary of these is the recognition by policy makers that markets are unstable in the context of uncertainty, and circuit breakers are unable to address the disruptive effects of market crashes that are not justified by economic news, i.e., do not reflect economic conditions correctly. This failure of market price setting should prompt more discussions about how market regulations can prevent crashes. From the point of view of news reporting, the result that market dynamics are unreliable indicators of economic conditions is also essential, as *post-hoc* justifications for market declines may unjustifiably assume fundamentally driven market prices. Absent improvements in policy, our methods may be used by investors either to assure themselves of market stability when indicators are not predicting crashes, or to anticipate market crashes. We note that we have not analyzed the financial benefits for strategies that involve selling securities prior to a crash and buying them at the time of a crash in anticipation of their subsequent increase. While this may be a successful strategy, alternatives exist. For example, for those who do not need to sell during a downturn, the history of panic induced market crashes suggests that holding securities may be a good strategy, as all of the market declines were followed by increases that restored value.

Finally, we note that the ability to distinguish between self-induced panic and the result of external effects may be widely applicable to collective behaviors [87], and can be applied more generally as an early warning signal that may anticipate sudden changes in the behavior of a wide range of complex systems.

## Methods

### Dynamic network model of daily stock returns

Consider a network representing an economic market with *N* variable nodes taking only the values −1 or 1, representing decreasing or increasing returns of a particular stock. In addition there are *D* and *U* nodes frozen in state −1 and 1 respectively. At each time step a variable node is selected at random; with probability 1 − *p* the node copies the state of one of its connected neighbors, and with probability *p* the state remains unchanged. The frozen nodes are interpreted as external perturbations with negative and positive effects on the returns. Analytically extending *D* and *U* to be real numbers enables modeling arbitrary strengths of external perturbations. A detailed account of the dynamic network model under external perturbations is given by [24] [25]. The model was first applied as a framework for early warning signals of real-world self-organized economic and market crises by [26]. Here we outline basic results that are pertinent to the study of self-organized market crises.

For a fully connected network the behavior of the system can be solved exactly as follows. The nodes are indistinguishable and the state of the network is fully specified by the number of nodes with internal state 1. Therefore, there are only *N* + 1 distinguishable global states, which we denote *σ*_*k*_, *k* = 0,1,…, *N*. The state *σ*_*k*_ has *k* variable nodes in state 1 and *N* − *k* variable nodes in state −1. If *P*_*t*_(*k*) is the probability of finding the network in the state *σ*_*k*_ at the time *t*, then *P*_*t*+1_(*k*) can depend only on *P*_*t*_(*k*), *P*_*t*_(*k* + 1) and *P*_*t*_(*k* − 1). The probabilities *P*_*t*_(*k*) define a vector of *N* + 1 components **P**_*t*_. In terms of **P**_*t*_ the dynamics is described by the equation

$$P_{t+1}=TP_{t}\equiv (1-\frac{\left(1-p\right)}{N(N+D+U-1)}A)P_{t}$$ (6)

where the time evolution matrix **T**, and also the auxiliary matrix **A**, is tri-diagonal. The non-zero elements of **A** are independent of *p* and are given by

$$\begin{array}{c} A_{k,k}=2k\left(N-k\right)+U\left(N-k\right)+Dk\\ A_{k,k+1}=-\left(k+1\right)\left(N+D-k-1\right)\\ A_{k,k-1}=-\left(N-k+1\right)\left(U+k-1\right). \end{array}$$ (7)

The transition probability from state *σ*_*M*_ to *σ*_*L*_ after a time *t* can be written as

$$P\left(L,t;M,0\right)=\sum_{r=0}^{N}b_{rM}a_{rL}\lambda_{r}^{t}\;.$$ (8)

where *a*_*rL*_ and *b*_*rM*_ are the components of the right and left *r*-th eigenvectors of the evolution matrix, **a**_*r*_ and **b**_*r*_. Thus, the dynamical problem has been reduced to finding the right and left eigenvectors and the eigenvalues of **T**.

The eigenvalues *λ*_*r*_ of **T** are given by

$$\lambda_{r}=1-\frac{\left(1-p\right)}{N(N+D+U-1)}r\left(r-1+D+U\right)$$ (9)

and satisfy 0 ≤ *p* ≤ *λ*_*r*_ ≤ 1. The equation for *P*(*L*, *t*;*M*,0) shows that the asymptotic state of the network is determined only by the right and left eigenvectors with unit eigenvalue, i.e., by the eigenvectors of *λ*_0_ = 1. The coefficients of the corresponding (unnormalized) left eigenvector are simply *b*_0*k*_ = 1. The coefficients *a*_0*k*_ of the right eigenvector are given by the Taylor expansion of the hypergeometric function *F*(−*N*, *U*,1 − *N* − *D*, *x*) ≡ ∑_*k*_ *a*_0*k*_ *x* ^*k*^. After normalization these coefficients give the stationary distribution

$$\rho (k)=\frac{\left(\begin{array}{c} U+k-1\\ k \end{array}\right)\left(\begin{array}{c} N+D-k-1\\ N-k \end{array}\right)}{\left(\begin{array}{c} N+D+U-1\\ N \end{array}\right)}.$$ (10)

This is the probability of finding the network with *k* nodes in state 1 at equilibrium and it is independent of the initial state. The other eigenvectors can also be calculated and are also related to hypergeometric functions.

**Fig 9:**
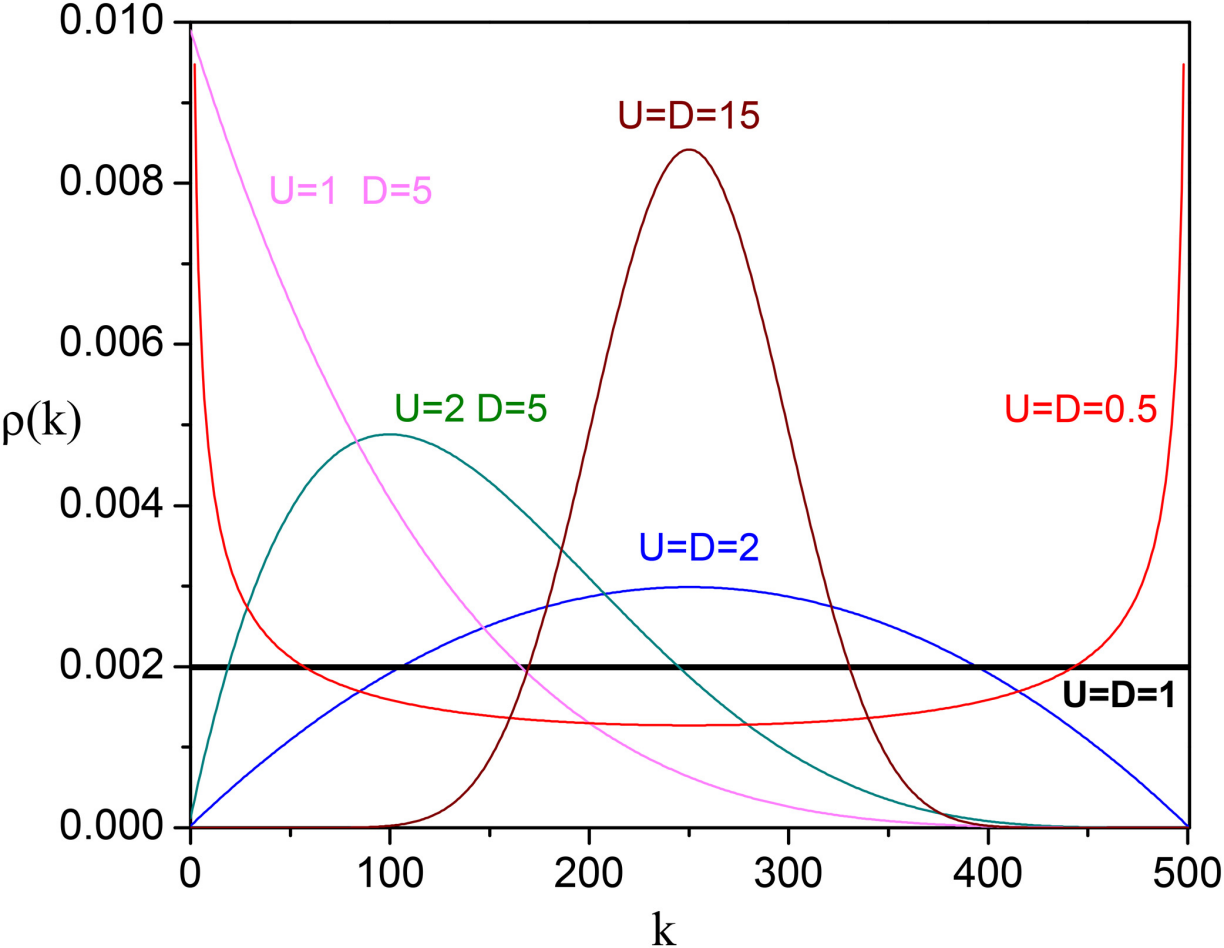
Stationary distributions for different values of *U* and *D*. Probability distributions of finding the network with *k* nodes in state 1 at equilibrium for different values of *U* and *D*. The number of variable nodes is *N* = 500.

We observe different types of behavior, which is characteristic of a first-order phase transition, that occur as we move around in the (*D*, *U*)-parameter space. Fig 9 shows examples of the distribution *ρ*(*k*) for a network with *N* = 500 and various values of *D* and *U*. One important feature of this solution is that for *D* = *U* = 1 we obtain *ρ*(*k*) = 1/(*N* + 1) for all values of *N*, i.e., *D* = *U* = 1 is the *critical value* of this model. Thus all states *σ*_*k*_ are equally likely and the system executes a random walk through the state space. In the limit *N* → ∞, *D* = *U* = 1 marks the transition between disordered and ordered states.

For *D*, *U* > 1, we obtain skewed unimodal distributions with peak at *U*/(*U* + *D*) corresponding to the fraction of stocks in the network that move up. The market is in the “up” phase if *U* > *D* or in the “down” phase if *U* < *D*. For *D*, *U* > > 1, *ρ*(*k*) resembles a Gaussian distribution (see derivation in [24] [25]) and if *D* = *U* about half the nodes are in state −1 and half in state +1, similarly to a magnetic material at high temperatures.

For *D*, *U* < 1—the bistable (hysteresis) region—we obtain bimodal distributions in which either of the two network phases can exist, similar to the magnetization state in the Ising model below the critical temperature. For *D* = *U* < < 1, the distribution peaks at all nodes −1 or all nodes +1, similar to a magnetized state at low temperatures.

Finally, for *U* > 1, *D* < 1 or *U* < 1, *D* > 1, we obtain unimodal distributions with peaks at all nodes +1 or all nodes −1, respectively.

As mentioned earlier (see Table 1), a significant drop in the value of the model parameter *U* (= *D*) occurred in the 2000–2002 period, followed by a plateau that declined gradually beginning in mid-2007 until it hit the critical value at *D* = *U* = 1. In other words, over the decade of the 2000s the probability of a co-movement fraction became progressively flatter, and in 2008 the likelihood of any fraction is almost the same for any value—this corresponds to the critical point of our model, before entering the hysteresis region.

It might seem that the critical point should depend on the size of the external influence relative to the number of nodes in the system, i.e., *U*/*N*. However, this is an order to disorder transition, and, as with the temperature in physics models of phase transitions, the critical value does not depend on the system size. For all values of *D* = *U*, the nodes have equal probability of being in state +1 or −1. Thus, each node experiences an environment that drives it equally toward positive and negative values. The role of the external influence is only as a perturbation promoting transitions between states of the distribution. In this context, even though the external influence on any one node decreases as *N* increases, the influence across all nodes is independent of *N*. This is because each node picks the external node to copy in proportion to 1/*N*. Thus, the average number of nodes that are changed per time step by the external influence is independent of *N*.

This system can model a number of situations. An example is the Ising model, where our dynamics are equivalent to Glauber dynamics [88] for small external magnetic fields (*h*) and all temperatures (*T*) including the phase transition regime, for uniform connectivity lattices in the thermodynamic limit. The Ising model parameters are *J*/*kT* → 1/(*z* + *D* + *U*) and *h*/*J* → (*U* − *D*), where *z* is the number of nearest neighbors and *J* the nearest-neighbor interaction strength. Relevant network structures include crystalline 3-D lattices and random networks for amorphous spin-glasses; fully connected networks correspond to long range interactions or the mean field approximation. The system can also model an election with two candidates where some of the voters have a fixed opinion while the rest change their intention according to the opinion of others. Another application is to epidemics that spread upon contact between infected nodes (e.g., individuals or computers), a case for which we would set *D* = 0 to study spreading dynamics. Finally, this model has also an analogue in population genetics and can be mapped exactly into the Wright-Fisher-Moran model with two alleles and mutation. [54] [55] Consider a population of *N* haploid individuals and a gene with alleles *A*_1_ and *A*_2_. Sexual reproduction occurs between random pairs of individuals with the offspring replacing one of the expiring parents. After the allele of the offspring is chosen with equal probability between the parents, there is also a probability *μ*_1_ to mutate from *A*_1_ to *A*_2_ or *μ*_2_ to mutate from *A*_2_ to *A*_1_. The number of alleles *A*_1_ in the population in equilibrium is given by Eq 10 with

$$U=\frac{2\mu_{2}\left(N-1\right)}{1-\mu_{1}-\mu_{2}}\;D=\frac{2\mu_{1}\left(N-1\right)}{1-\mu_{1}-\mu_{2}}.$$ (11)

This problem was first considered by Watterson and Gladstein [89, 90] with no mutation and latter generalized by Cannings [91]. A detailed account is given by de Aguiar and Bar-Yam [55].

Although the positive-return distribution given by Eq 10 is obtained assuming fully connected networks, here we show that our exact results are excellent approximations for other networks, including random, regular lattice, scale-free, and small world networks [24, 25]. These approximations can be useful, for example, if our model is applied to a network constructed based on the cross-correlations between pairs of stock-price time series [11–18]. For these networks, which are not fully connected, the effect of the frozen nodes is amplified and can be quantified as follows: the probability that a free node copies a frozen node is *P*_*i*_ = (*D* + *U*)/(*D* + *U* + *k*_*i*_) where *k*_*i*_ is the degree of the node. For fully connected networks *k*_*i*_ = *N* − 1 and we obtain *P*_*FC*_ ≡ (*D* + *U*)/(*D* + *U* + *N* − 1). For general networks an average value *P*_*av*_ can be calculated by replacing *k*_*i*_ by the average degree *k*_*av*_ = 1/*N*∑_*i*_ *k*_*i*_. We can then define effective numbers of frozen nodes, *D*_*ef*_ and *U*_*ef*_, as being the values of *D* and *U* in *P*_*FC*_ for which *P*_*av*_ ≡ *P*_*FC*_. This leads to

$$D_{ef}=fD,\;\;U_{ef}=fU$$ (12)

where *f* = (*N* − 1)/*k*_*av*_. Therefore, as the network acquires more internal connections and *k*_*av*_ increases, the effective values of *D* and *U* decrease. For well behaved distributions, corrections involving higher moments can be obtained by integrating *P*_*i*_ times the degree distribution and expanding around *k*_*av*_.

**Fig 10:**
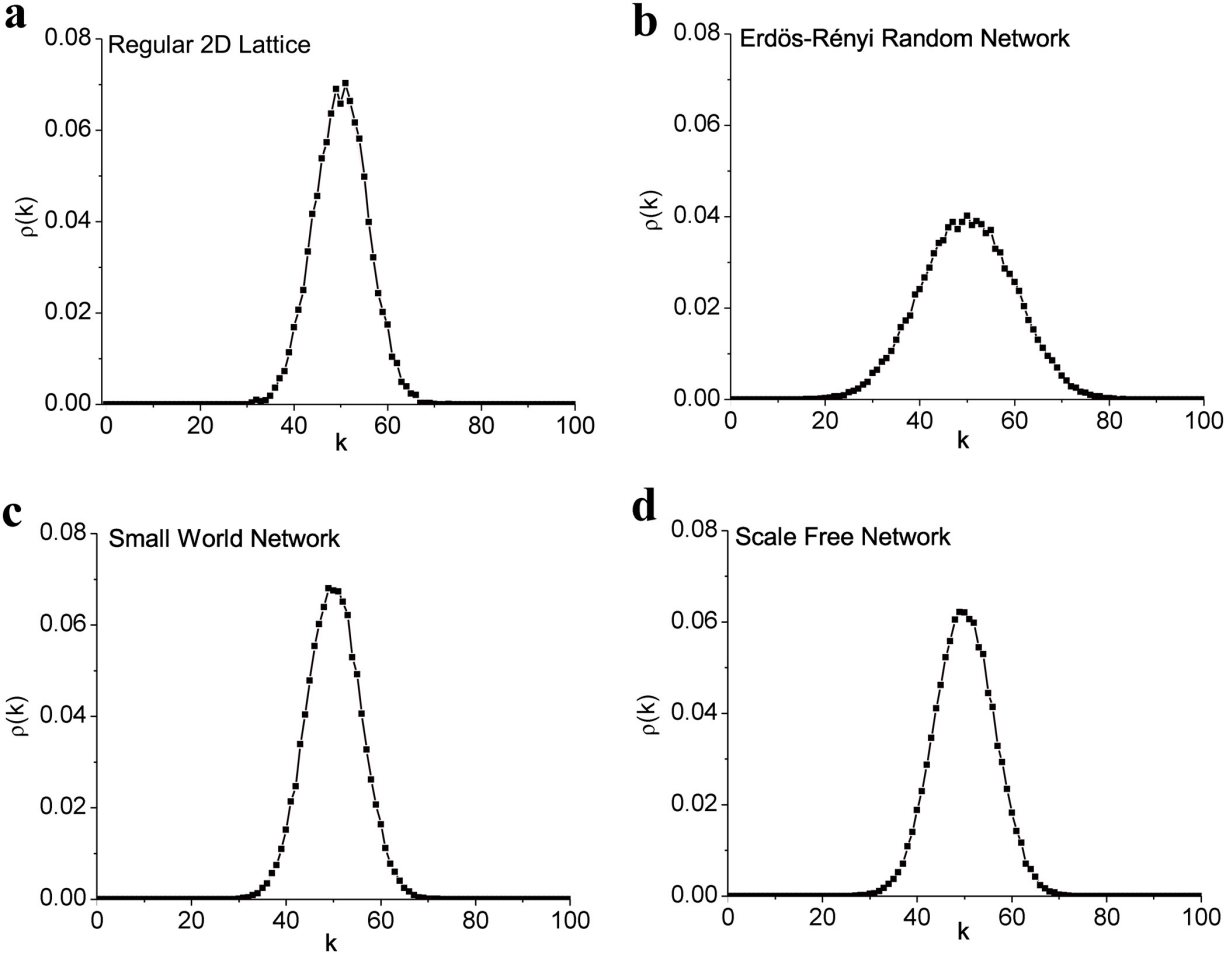
Asymptotic probability distribution for networks with different topologies. In all cases *N* = 100, *D* = *U* = 5, *t* = 10,000, and the number of realizations is 50,000. The theoretical curve is drawn with effective numbers of frozen nodes *D*_*ef*_ and *U*_*ef*_: (a) regular 2-D lattice *D*_*ef*_ = *U*_*ef*_ = 150; (b) Erdös-Rényi random network *D*_*ef*_ = *U*_*ef*_ = 17; (c) small world network *D*_*ef*_ = *U*_*ef*_ = 143; (d) scale-free *D*_*ef*_ = *U*_*ef*_ = 80.

Fig 10 shows examples of the equilibrium distribution attained by networks with different topologies. Panel (a) shows the probability distribution for a 2-D regular lattice with 10 × 10 nodes. The theoretical result is given by Eq 10 but for *D*_*ef*_ = *U*_*ef*_ = 150, which is of the order of 99*D*/4, where 99 is the number of neighbors in the fully connected case and 4 the number of neighbors in the regular lattice. The larger effective values of *D* and *U* in this case are easy to understand: the weaker propagation of the perturbations resulting from the smaller connectivity is compensated by an increase in the effective size of the perturbation. Panel (b) shows the probability distribution for an Erdös-Rényi random network with connection probability between nodes of *pc* = 0.3 (nodes have 30 connections each on the average). This time the theoretical result fits the curve only if *D*_*ef*_ = *U*_*ef*_ = 17 ≈ *D*/*pc*. Panel (c) shows a small world version of the regular lattice [92], where 30 connections were randomly re-connected, creating shortcuts between otherwise distant nodes. The average number of connections per node is the same as in the regular lattice, but the effective size of the perturbations decreases to *D*_*ef*_ = *U*_*ef*_ = 143, since the shortcuts promote faster propagation. Finally, for a scale-free network (panel (d)) grown from an initial cluster of 6 nodes adding nodes with 3 connections each following the preferential attachment rule [93], the effective values of *D* and *U* are 80. Since the average number of connections per node in this network is close to 3, the linear rule applied for the random and regular networks would result in *D*_*ef*_ = *U*_*ef*_ = 165. Thus the scale-free topology plays an important role in propagating the perturbations more effectively than in regular networks. The fit of equilibrium distributions by effective values presented in Fig 10 holds for unequal values of *D* and *U*.

### Curve fits

Theoretical fits are computed from an unbiased estimator of the standard deviation. The distribution takes values *k* = 0,…, *N*. We are interested in the positive fraction, or *k*/*N*, rather than the number of positive nodes. The central moments of the positive fraction distribution can be computed from Eq 10. We express the mean, *c*_1_, and variance, *c*_2_ in terms of *ξ* = *U*/(*U* + *D*) and *a* = *U* + *D*.

$$\begin{array}{ccc} c_{1} & = & \xi \end{array}$$ (13)

$$\begin{array}{ccc} c_{2} & = & \frac{\xi (1-\xi )(1+a/N)}{a+1} \end{array}$$ (14)

Eqs 13 and 14 can be inverted to solve for *ξ* and *a*:

$$\begin{array}{ccc} \xi & = & c_{1} \end{array}$$ (15)

$$\begin{array}{ccc} a & = & \frac{c_{1}\left(1-c_{1}\right)-c_{2}}{c_{2}-c_{1}\left(1-c_{1}\right)/N} \end{array}$$ (16)

For the case of stocks, fits using *c*_1_ = 0.5 are better fits as measured by the *χ* ^2^ goodness-of-fit test than fits achieved by setting *c*_1_ to the mean of the empirical distribution.

### Data sources

To compute empirical distributions, we used daily returns from the Russell 3000, restricted to stocks trading on the NYSE, NYSE Alternext, Nasdaq Capital, and Nasdaq Stock markets. The Russell 3000 is maintained by Russell Investments, and is reconstrustructed every twelve months, with the new composition announced near the end of June. It is highly correlated with the S&P 500 index. The Russell 3000 and specific details of the selection process may be obtained from Russell Investments [94]. All the historical return data is publicly available from Yahoo, Google and other online sources, including Capital IQ [95], which we used for this purpose.

To compute the empirical distribution of the positive-return fraction, we used two methods. For the period from July 1999 to June 2010, we retrieved daily returns of large-cap stocks from the Russell 3000 membership lists, published at the end of June for the years 1999 through 2009. Daily returns of the stocks on the list were retrieved for the following twelve month period beginning in July. Stocks that were delisted during this period were included for all days before delisting. For the period before July 1999, we combined ticker symbols from the Russell 3000 membership lists from June 2001, 2004, and 2007, and retrieved daily returns for the symbols back to 1985. Each positive return fraction was computed with more than 140 stocks (overlapping heavily with the S&P 500 index).

## References

[1] Goldberger AS. Structural equation methods in the social sciences. Econometrica. 1972; 40: 979–1001. 10.2307/1913851

[2] Eichengreen B, Rose AK, Wyplosz C. Exchange market mayhem: the antecedents and aftermath of speculative attacks. Am Econ J Econ Policy. 1995; 10: 251–312.

[3] Kaminsky G, Lizondo S, Reinhart CM. Leading indicators of currency crises. Staff Pap Int Monet Fund. 1998; 45: 1–48. 10.2307/3867328

[4] Berg A, Pattillo C. Predicting currency crises: the indicators approach and an alternative. J Int Money Financ. 1999; 18: 561–586. 10.1016/S0261-5606(99)00024-8

[5] Borio C, Lowe P. Asset prices, financial and monetary stability: exploring the nexus; 2002. Preprint. Available: SSRN 846305. Accessed 31 May 2015.

[6] Bordo MC, Jeanne O. Monetary policy and asset prices: does ‘benign neglect’ make sense? International Finance. 2002; 5: 139–164.

[7] Adrian T, Shin HS. Money, liquidity, and monetary policy. Am Econ Rev. 2009; 99: 600–605. 10.1257/aer.99.2.600

[8] Kritzman M, Li Y, Page S, Rigobon R. Principal components as a measure of systemic risk. J Portfolio Manage. 2011; 37: 112–126. 10.3905/jpm.2011.37.4.112

[9] Morris G. The complete guide to market breadth indicators: how to analyze and evaluate market direction and strength. 1st ed New York, NY: McGraw-Hill; 2005.

[10] Rose AK, Spiegel MM. Cross-country causes and consequences of the crisis: an update. Eur Econ Rev. 2011; 55: 309–324. 10.1016/j.euroecorev.2010.12.006

[11] Mantegna RN, Stanley HE. An introduction to econophysics. 1st ed Cambridge: Cambridge University Press; 2000.

[12] Mantegna RN. Hierarchical structure in financial markets. Eur Phys J B. 1999; 11: 193–197. 10.1007/s100510050929

[13] Vandewalle N, Brisbois F, Tordoir X. Self-organized critical topology of stock markets; 2000. Preprint. Available: arXiv:cond-mat/0009245. Accessed 31 May 2015.

[14] Bonnanno G, Caldarelli G, Lillo F, Mantegna RN. Topology of correlation-based minimal spanning trees in real and model markets. Phys Rev E Stat Nonlin Soft Matter Phys. 2003; 68: 046103 10.1103/PhysRevE.68.046130 14683025

[15] Onnela JP, Chakraborti A, Kaski K, Kertesz J, Kanto A. Dynamics of market correlations: taxonomy and portfolio analysis. Phys Rev E Stat Nonlin Soft Matter Phys. 2003; 68: 056110 10.1103/PhysRevE.68.056110 14682849

[16] Harmon D, Stacey B, Bar-Yam Y, Bar-Yam Y. Networks of economic market interdependence and systemic risk; 2010. Preprint. Available: arXiv:1011.3707v2. Accessed 31 May 2015.

[17] Kenett DY, Preis T, Gur-Gershgoren G, Ben-Jacob E. Dependency network and node influence: application to the study of financial markets. Int J Bifurcat Chaos. 2012; 22: 1250181 10.1142/S0218127412501817

[18] Kenett DY, Tumminello M, Madi A, Gur-Gershgoren G, Mantegna RN, Ben-Jacob E. Dominating clasp of the financial sector revealed by partial correlation analysis of the stock market. PLoS One. 2010; 5: e15032 10.1371/journal.pone.0015032 21188140 PMC3004792

[19] LeBaron B, Arthur WB, Palmer R. Time series properties of an artificial stock market. J Econ Dyn Control. 1999; 23: 1487–1516. 10.1016/S0165-1889(98)00081-5

[20] Levy M, Levy H, Solomon S. Microscopic simulation of financial markets: from investor behavior to market phenomena. 1st ed San Diego: Academic Press; 2000.

[21] Hommes C, Wagener F. Complex evolutionary systems in behavioral finance In: Hens T, Schenk-Hoppá KR, editors. Handbook of financial markets: dynamics and evolution. Amsterdam: North Holland; 2009 pp. 217–277.

[22] Lux T, Marchesi M. Scaling and criticality in a stochastic multi-agent model of a financial market. Nature. 1999; 397: 498–500. 10.1038/17290

[23] Lagi M, Bar-Yam Y, Bertrand K, Bar-Yam Y. The food crises: a quantitative model of food prices including speculators and ethanol conversion; 2011. Preprint. Available: arXiv:1109.4859. Accessed 31 May 2015.

[24] Chinellato DD, de Aguiar MAM, Epstein IR, Braha D, Bar Yam Y. Dynamical response of networks under external perturbations: exact results; 2007. Preprint. Available: arXiv:0705.4607v2. Accessed 13 February 2015.

[25] Chinellato DD, Epstein IR, Braha D, Bar Yam Y, de Aguiar MAM. Dynamical response of networks under external perturbations: exact results. J Stat Phys. 2015; 159: 221–230. 10.1007/s10955-015-1189-x

[26] Harmon D, de Aguiar MAM, Chinellato DD, Braha D, Epstein IR, Bar-Yam Y. Predicting economic market crises using measures of collective panic; 2011. Preprint. Available: arXiv:1102.2620. Accessed 31 May 2015. 10.1371/journal.pone.0131871 PMC4506134 26185988

[27] Majdandzic A, Podobnik B, Buldyrev SV, Kenett DY, Havlin S, Stanley HE. Spontaneous recovery in dynamical networks. Nat Phys. 2014; 10: 34–38. 10.1038/nphys2819

[28] Bury T. Predicting trend reversals using market instantaneous state. Physica A. 2014; 404: 79–91. 10.1016/j.physa.2014.02.044

[29] Preis T, Stanley HE. Switching phenomena in a system with no switches. J Stat Phys. 2010; 138: 431–446. 10.1007/s10955-009-9914-y

[30] Sornette D, Johansen A, Bouchaud JP. Stock market crashes, precursors and replicas. J Phys I. 1996; 6: 167–175.

[31] Feigenbaum JA, Freund PGO. Discrete scale invariance in stock markets before crashes. Int J Mod Phys B. 1996; 10: 3737–3745. 10.1142/S021797929600204X

[32] Sornette D, Johansen A. Large financial crashes. Physica A. 1997; 245: 411–422. 10.1016/S0378-4371(97)00318-X

[33] Sornette D, Stauffer D, Takayasu H. Market fluctuations II: multiplicative and percolation models, size effects, and predictions In: Bunde A, Kropp J, Schellnhuber HJ, editors. The science of disasters: climate disruptions, heart attacks, and market crashes. New York, NY: Springer; 2002 pp. 411–433.

[34] Sornette D. Endogenous versus exogenous origins of crises In: Albeverio S, Jentsch V, Kantz H, editors. Extreme events in nature and society. New York, NY: Springer; 2006 pp. 95–116.

[35] Preis T, Reith D, Stanley HE. Complex dynamics of our economic life on different scales: insights from search engine query data. Philos Trans R Soc Lond A. 2010; 368: 5707–5719. 10.1098/rsta.2010.0284 21078644

[36] Ross SA. The arbitrage theory of capital asset pricing. J Econ Theory. 1976; 13: 341–360. 10.1016/0022-0531(76)90046-6

[37] Chamberlain G, Rothschild M. Arbitrage, factor structure, and mean-variance analysis on large asset markets. Econometrica. 1983; 51: 1281–1304. 10.2307/1912275

[38] Smith RD. The spread of the credit crisis: view from a stock correlation network. J Korean Phys Soc. 2009; 54: 2460–2463. 10.3938/jkps.54.2460

[39] Jorion P. Value at Risk: the new benchmark for managing financial risk. 3rd ed New York, NY: McGraw-Hill; 2006.

[40] Shapira Y, Kenett DY, Raviv O, Ben-Jacob E. Hidden temporal order unveiled in stock market volatility variance. AIP Adv. 2011; 1: 022127 10.1063/1.3598412

[41] Christie AA. The stochastic behavior of common stock variances: value, leverage and interest rate effects. J financ econ. 1982; 10: 407–432. 10.1016/0304-405X(82)90018-6

[42] Nelson D. Conditional heteroskedasticity in asset returns: a new approach. Econometrica. 1991; 45: 347–370. 10.2307/2938260

[43] Bekaert G, Wu G. Asymmetric volatility and risk in financial markets. Rev Financ Stud. 2000; 13: 1–42. 10.1093/rfs/13.1.1

[44] Wu G. The determinants of asymmetric volatility. Rev Financ Stud. 2001; 14: 837–859. 10.1093/rfs/14.3.837

[45] Braha D, Bar-Yam Y. From centrality to temporary fame: dynamic centrality in complex networks. Complexity. 2006; 12: 59–63. 10.1002/cplx.20156

[46] Hill SA, Braha D. Dynamic model of time-dependent complex networks. Phys Rev E Stat Nonlin Soft Matter Phys. 2010; 82: 046105 10.1103/PhysRevE.82.046105 21230343

[47] Garas A, Argyrakis P, Havlin S. The structural role of weak and strong links in a financial market network. Eur Phys J B. 2008; 63: 265–271. 10.1140/epjb/e2008-00237-3

[48] Schweitzer F, Fagiolo G, Sornette D, Vega-Redondo F, Vespignani A, White DR. Economic networks: the new challenges. Science. 2009; 325: 422–425. 19628858 10.1126/science.1173644

[49] Emmert-Streib F, Dehmer M. Influence of the time scale on the construction of financial networks. PLoS One. 2010; 5: e12884 10.1371/journal.pone.0012884 20949124 PMC2948017

[50] Guillaume DM, Dacorogna MM, Davé RR, Müller UA, Olsen RB, Pictet OV. From the bird’s eye to the microscope: a survey of new stylized facts of the intra-daily foreign exchange markets. Financ Stoch. 1997; 1: 95–129. 10.1007/s007800050018

[51] Gopikrishnan P, Meyer M, Amaral LAN, Stanley HE. Inverse cubic law for the distribution of stock price variations. Eur Phys J B. 1998; 3: 139–140. 10.1007/s100510050292

[52] Kardar M, Parisi G, Zhang YC. Dynamic scaling of growing interfaces. Phys Rev Lett. 1986; 56: 889 10.1103/PhysRevLett.56.889 10033312

[53] Kardar M. Statistical physics of fields. 1st ed Cambridge: Cambridge University Press; 2007.

[54] Ewens WJ. Mathematical Population Genetics I Theoretical Introduction. 1st ed New York, NY: Springer Verlag; 1979.

[55] de Aguiar MAM, Bar-Yam Y. Moran model as a dynamical process on networks and its implications for neutral speciation. Phys Rev E Stat Nonlin Soft Matter Phys. 2011; 84: 031901 10.1103/PhysRevE.84.031901 22060397

[56] Arthur WB. Self-reinforcing mechanisms in economics In: Anderson PW, Arrow KJ, Pines D, editors. The economy as an evolving complex system. New York, NY: Westview Press; 1988 pp. 9–33.

[57] Wolfenstein M. Disaster. 1st ed Glencoe, Illinois: Free Press; 1957.

[58] Smelser NJ. Theory of Collective Behavior. 1st ed Glencoe, Illinois: Free Press; 1963.

[59] Quarantelli EL. The sociology of panic In: Smelser NJ, Baltes PB, editors. International Encyclopedia of the Social and Behavioral Sciences. New York, NY: Elsevier; 2001 pp. 11020–11023.

[60] Mawson AR. Understanding mass panic and other collective responses to threat and disaster. Psychiatry. 2005; 68: 95–113. 16247853 10.1521/psyc.2005.68.2.95

[61] Diamond DW, Dybvig PH. Bank runs, deposit insurance, and liquidity. J Polit Econ. 1983; 91: 401–419. 10.1086/261155

[62] Calomiris CW, Gorton G. The origins of banking panics: models, facts, and bank regulation In: Hubbard RG, editor. Financial markets and financial crises. Chicago: National Bureau of Economic Research; 1990 pp. 109–175.

[63] Cutler D, Poterba J, Summers L. What moves stock prices? J Portfolio Manage. 1989; 15: 4–12. 10.3905/jpm.1989.409198

[64] Mann L, Nagel T, Dowling P. A study of economic panic: The “run” on the Hindmarsh Building Society. Sociometry. 1976; 39: 223–235. 10.2307/2786515 1006359

[65] Galbraith JK. The Great Crash 1929. 1st ed New York, NY: Houghton Mifflin; 1954.

[66] Kindleberger C. Manias, panics, and crashes. 1st ed New York, NY: John Wiley & Sons Inc; 1978.

[67] Feigenbaum JA. A statistical analysis of log-periodic precursors to financial crashes. Quant. Finance. 2001; 1: 346–360. 10.1080/713665875

[68] Sornette D, Johansen A. Significance of log-periodic precursors to financial crashes. Quant. Finance. 2001; 1: 452–471. 10.1088/1469-7688/1/4/305

[69] Bree DS, Joseph NL. Fitting the log periodic power law to financial crashes: a critical analysis; 2010. Preprint. Available: arXiv:1002.1010v1. Accessed 31 May 2015.

[70] Cho A. Econophysics: still controversial after all these years. Science. 2009; 325: 408 10.1126/science.325_408 19628851

[71] Scheffer M, Bascompte J, Brock WA, Brovkin V, Carpenter SR, Dakos V, et al Early-warning signals for critical transitions. Nature. 2009; 461: 53–59. 10.1038/nature08227 19727193

[72] Scheffer M, Carpenter SR, Lenton TM, Bascompte J, Brock W, Dakos V, et al Anticipating critical transitions. Science. 2012; 338: 344–348. 10.1126/science.1225244 23087241

[73] De Long JB, Shleifer A, Summers LH, Waldmann RJ. Positive feedback investment strategies and destabilizing rational speculation. J Finance. 1990; 45: 379–395. 10.1111/j.1540-6261.1990.tb03695.x

[74] De Long JB, Shleifer A, Summers LH, Waldmann RJ. Noise trader risk in financial markets. J Polit Econ. 1990; 98: 703–738. 10.1086/261703

[75] Scharfstein DS, Stein JC. Herd behavior and investment. Am Econ Rev. 1990; 80: 465–479.

[76] Bikhchandani S, Hirshleifer D, Welch I. Learning from the behavior of others: conformity, fads, and informational cascades. J Econ Perspect. 1998; 12: 151–170. 10.1257/jep.12.3.151

[77] Dow Jones Averages. Dow Jones Industrial Average, Milestones. Available: http://www.djaverages.com/?view=industrial&page=milestones.

[78] Carlson M. A brief history of the 1987 stock market crash with a discussion of the federal reserve response. Washington, DC: Finance and Economic Discussion Series, Divisions of Research & Statistics and Monetary Affairs Federal Reserve Board; 2007.

[79] Giancarlo C, Pesenti P, Roubini N. What caused the Asian currency and financial crisis? Japan World Econ. 1999; 11: 305–373.

[80] Grech D, Mazur Z. Can one make any crash prediction in finance using the local Hurst exponent idea? Physica A. 2004; 336: 133–145.

[81] Sornette D. Why Stock Markets Crash. 1st ed Princeton: Princeton University Press; 2003.

[82] Longin F, Solnik B. Extreme correlation of international equity markets. J Finance. 2001; 56: 649–676. 10.1111/0022-1082.00340

[83] Forbes KJ, Rigobon R. No contagion, only interdependence: measuring stock market comovements. J Finance. 2002; 57: 2223–2261. 10.1111/0022-1082.00494

[84] Caporale GM, Cipollini A, Spagnolo N. Testing for contagion: a conditional correlation analysis. J of Empirical Finance. 2005; 12: 476–489. 10.1016/j.jempfin.2004.02.005

[85] Fawcelt T. An Introduction to ROC analysis. Pattern Recognit Lett. 2006; 27: 861–874. 10.1016/j.patrec.2005.10.010

[86] Braha D, Shmilovici A. Data mining for improving a cleaning process in the semiconductor industry. IEEE Trans Semicond Manuf. 2002; 15: 91–101. 10.1109/66.983448

[87] Bar Yam Y. Dynamics of complex systems. 1st ed Reading: Perseus Press; 1997.

[88] Glauber RJ. Time-dependent statistics of the Ising model. J Math Phys. 1963; 4: 294–307. 10.1063/1.1703954

[89] Watterson GA. Markov chains with absorbing states: a genetic example. Ann Math Statist. 1961; 32: 716–729. 10.1214/aoms/1177704967

[90] Gladstein K. The characteristic values and vectors for a class of stochastic matrices arising in genetics. SIAM J Appl Math. 1978; 34: 630–642. 10.1137/0134050

[91] Cannings C. The latent roots of certain Markov chains arising in genetics; a new approach. I. Haploid models. Adv Appl Prob. 1974; 6: 260–290. 10.2307/1426293

[92] Watts DJ, Strogatz SH. Collective dynamics of ‘small-world‘networks. Nature. 1998; 393: 440–442. 10.1038/30918 9623998

[93] Albert R, Barabási AL. Statistical mechanics of complex networks. Rev Mod Phys. 2002; 74: 47–97. 10.1103/RevModPhys.74.47

[94] Russell Investments. Russell U.S. Equity Indexes Construction and Methodology. Available: http://www.russell.com/indexes/documents/Methodology.pdf.

[95] S&P Capital IQ. Available: https://www.capitaliq.com.

